# Reevaluating age restrictions of spinal metastasis surgery in elderly groups with over 2-year follow-up

**DOI:** 10.1007/s10143-023-02217-8

**Published:** 2023-11-21

**Authors:** Pavlina Lenga, Philip Dao Trong, Vassilios Papakonstantinou, Karl Kiening, Andreas W. Unterberg, Basem Ishak

**Affiliations:** Department of Neurosurgery, Heidelberg University Hospital, Im Neuenheimer Feld 400, 69120 Heidelberg, Germany

**Keywords:** Spinal metastasis, Epidural spinal cord compression, Surgery, Comorbidities, Age

## Abstract

This study aimed to compare and assess clinical outcomes of spinal metastasis with epidural spinal cord compression (MESCC) in patients aged 65–79 years and ≥ 80 years with an acute onset of neurological illness who underwent laminectomy. A second goal was to determine morbidity rates and potential risk factors for mortality. This retrospective review of electronic medical records at a single institution was conducted between September 2005 and December 2020. Data on patient demographics, surgical characteristics, complications, hospital clinical course, and 90-day mortality were also collected. Comorbidities were assessed using the age-adjusted Charlson comorbidity index (CCI). A total of 99 patients with an overall mean age of 76.2 ± 3.4 years diagnosed with MESCC within a 16-year period, of which 65 patients aged 65–79 years and 34 patients aged 80 years and older were enrolled in the study. Patients aged 80 and over had higher age-adjusted CCI (9.2 ± 2.1) compared to those aged 65–79 (5.1 ± 1.6; *p* < 0.001). Prostate cancer was the primary cause of spinal metastasis. Significant neurological and functional decline was more pronounced in the older group, evidenced by Karnofsky Performance Index (KPI) scores (80+ years: 47.8% ± 19.5; 65–79 years: 69.0% ± 23.9; *p* < 0.001). Despite requiring shorter decompression duration (148.8 ± 62.5 min vs. 199.4 ± 78.9 min; *p* = 0.004), the older group had more spinal levels needing decompression. Median survival time was 14.1 ± 4.3 months. Mortality risk factors included deteriorating functional status and comorbidities, but not motor weakness, surgical duration, extension of surgery, hospital or ICU stay, or complications. Overcoming age barriers in elderly surgical treatment in MSCC patients can reduce procedural delays and has the potential to significantly improve patient functionality. It emphasizes that age should not be a deterrent for spine surgery when medically necessary, although older MESCC patients may have reduced survival.

## Introduction

As diagnostic techniques and therapeutic modalities advance in the realm of oncology, the longevity of patients suffering from cancer has been substantially augmented [1–3]. Among the myriad of sites for metastasis, the spine is a common target, predominantly affecting the bone structures of the vertebrae with an incidence rate between 60 and 70% [4]. However, spinal metastasis with epidural spinal cord compression (MESCC) presents in only 5–10% of these cases [4]. Such cases can induce symptoms ranging from lower back pain to neurological deterioration, encompassing motor weakness and bowel or bladder dysfunction, thereby necessitating urgent surgical intervention.

Previous literature advocates surgical decompression or decompression combined with instrumentation as key strategies to maintain mobility in patients; however, multiple variables must be appraised prior to a definitive decision for surgical intervention [5–7]. Among these variables, patient age serves as a crucial factor due to the balancing act between surgical risks, life expectancy, and quality of life as surgical justification [8]. It is noteworthy that clinicians may approach aggressive surgical and oncological therapies with caution, potentially leading to suboptimal treatment [9]. A recent retrospective study on 34 octogenarians with acute neurological deterioration from MESCC demonstrated that emergency surgical decompression, even in a potentially unstable spine, had beneficial functional outcomes despite notable mortality rates ranging from 6 to 10% [10]. Despite these findings, there is still a lack of solid evidence on older patients, specifically those aged over 65, evaluating the potential benefits of surgical intervention in an acute setting. One of the critical questions pertains to establishing a cut-off age beyond which surgical management might not be offered due to the compromised physiological reserve of older patients.

In light of this gap in clinical evidence, the current study aims to assess and compare the clinical trajectory, and determine morbidity and mortality rates, in older patients aged between 65 and 79 years versus those aged 80 and above with acute onset MESCC undergoing microsurgical decompression in a potentially unstable spine. Additionally, we endeavor to assess the extent to which age might serve as a contraindication to surgery.

## Methods

### Study design, inclusion, and exclusion criteria

Data collection for this study involved a retrospective examination of clinical and imaging data, spanning the period between September 2005 and December 2020. This data was sourced from the institutional database, and the study was conducted in line with the Declaration of Helsinki. Our institution’s local ethics committee granted approval for the study (approval number 880/2021), and the necessity for informed consent was dismissed due to the study’s retrospective design.

The participants included in this study were patients aged 65–79 years and 80 years or older, confirmed to have thoracic and lumbar MESCC through histological examinations. Spinal stability was ascertained using magnetic resonance imaging (MRI) and computed tomography (CT), and two expert physicians, a neuroradiologist and neurosurgeon, employed the Spinal Instability Neoplastic Score (SINS) as an additional stability assessment tool. This score appraises tumor-related instability by accumulating scores for spinal location, lesion bone quality, pain, radiographic alignment, vertebral body collapse, and the posterolateral involvement of spinal elements. This was calculated for each patient. For the morphological evaluation of MESCC, the 6-point Epidural Spinal Cord Compression (ESCC) scale was employed, based on a consensus decision from three independent raters examining preoperative imaging. The study excluded patients under 65 years old, those with concurrent cervical pathology, and those with incomplete data. Furthermore, cases presenting with definitive spinal instability indicated by computed tomography were excluded, specifically those exhibiting bony deconstruction causing kyphosis or subluxation of the vertebral column, vertebral collapse exceeding 50% or bone necrosis, and complete loss of disc height. The presence of spinal instability often necessitates an instrumentation surgery, which is associated with extended surgical durations, prolonged recovery times stemming from increased muscular trauma, among other factors. Given these considerations and our objective to minimize potential biases in our study, we opted to exclude such patients. A total of 27 patients met this exclusion criterion. Additionally, seven patients were omitted due to inadequate documentation.

### Medical records

The data examined included patient demographics, comorbidities, American Society of Anesthesiologists scores (ASA), surgical duration, treated spinal levels, peri- and postoperative complications, ICU stay, hospital length of stay, readmission, reoperation, and mortality. This was sourced from the patients’ electronic records. Age-adjusted Charlson Comorbidity Index (CCI) was employed to assess preoperative comorbidities [11, 12]. The CCI was calculated for each patient and classified as no comorbidity (CCI = 0), minimal comorbidity (CCI = 1 or 2), moderate comorbidity (CCI = 3–5), or severe comorbidity (CCI > 5). Pre-treatment neurological conditions were evaluated using the Motor Score (MS) of the American Spinal Injury Association impairment grading system. Post-treatment MS data were derived from the last documented clinical encounter. The Karnofsky Performance Index (KPI) was utilized to assess changes in a patient’s condition, attributing grades to their ability to perform certain tasks according to the following scores: 100, normal state, no complaints; 70, inability to perform daily life activities; 50, requires significant assistance; 40, disability; 30, mandatory hospitalization; and 0, deceased. The extent of spinal injury was evaluated using the Frankel Grade (FG).

### Decision-making and surgical procedures

**Fig. 1:**
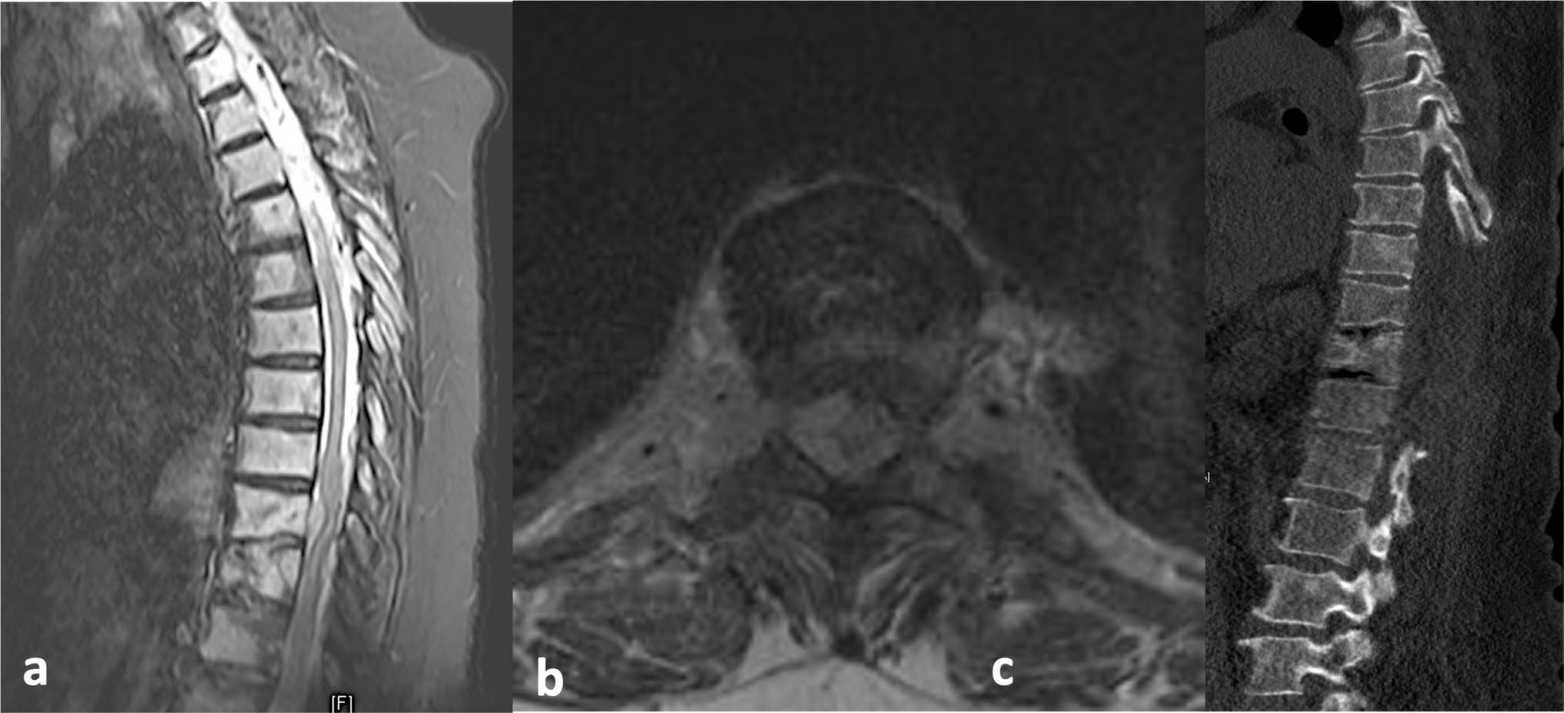
A 72-year-old male patient with multiple myeloma, displaying progressive lower extremity weakness. Sagittal (**a**) and axial (**b**) T2-weighted magnetic resonance imaging illustrates metastatic involvement of the T9 vertebral body. The vertebral body exhibits less than a 50% collapse. Postoperative CT scans (**c**) reveal a laminectomy performed at levels Th8–Th12, with complete decompression of the epidural space

All the included patients presented with acute neurological decline and underwent posterior decompression via laminectomy within the first 24 h of admission. The final decision on treatment plans was made by the attending spine surgeon, following comprehensive discussions with the experienced treatment team, considering the patient’s presenting neurological status, underlying pathologies, extent of the pathology, and disease prognosis. Routine clinical and radiological follow-up examinations were carried out before discharge and at 3 months postoperatively. An MRI of the spinal cord was performed to evaluate for potential tumor recurrence (Fig. 1).

### Surgical considerations

The operations were executed in line with our institutional guidelines, as previously described [10]. Two seasoned surgeons (KK and BI) conducted all procedures, with patients under full anesthesia. Patients were placed in a prone position on the surgical table. An incision along the midline provided access to the bone structures. Midline dissection continued using monopolar electrocautery. Spinous processes were excised, followed by a high-speed drill-assisted partial laminectomy. This drill was also used to refine the remaining lamina, and the laminectomy was finalized with angled instruments. Mostly coagulated blood was cleared, with hemostasis achieved without significant challenges. Subsequently, the tumor was either fully or partially excised by internal debulking or segmental removal, post-comprehensive hemostasis, and careful separation. Dural sac decompression was confirmed upon completion. Postoperatively, patients were encouraged to move, given the perceived stability of the spinal cord. Prolonged bed rest was not recommended for any of the cases reviewed.

### Statistical analysis

Categorical variables are delineated as counts and percentages, while continuous variables are expressed as means along with their standard deviations. The distribution normality of continuous variables was assessed through the Shapiro-Wilk test. Comparison of baseline characteristics, surgical duration, treated spinal levels, peri- and postoperative complications, length of stay (LOS), ICU duration, readmissions, reoperations, and mortality was conducted across groups. Independent *t*-tests were used for continuous variables and chi-squared tests for categorical variables. Changes in neurological status (MS) and functional outcomes (KPI) at the point of discharge were evaluated via the Wilcoxon rank test. In the subsequent analysis phase, binary logistic regression was employed to identify the risk factors contributing to the loss of ambulation. The threshold for statistical significance was established at a *p*-value equal to or less than 0.05.

## Results

### Demographics of patients

A total of 99 patients diagnosed with MESCC, including 65 patients aged 65–79 years and 34 patients aged 80 years and older, were enrolled in the study over a span of 16 years. The majority of the cohort were males (70.7%) with a mean age of 76.2 ± 3.4 years. Patients aged 80 and above presented a significantly higher age-adjusted CCI (9.2 ± 2.1) compared to their younger counterparts (5.1 ± 1.6; *p* < 0.001). No notable differences were observed between the groups concerning the location of MESCC. Prostate cancer-related spinal metastasis was most prevalent in both groups (65–79 years: *n* = 19, 29.2%; 80 years and older: *n* = 11, 32.4%; *p* = 0.077). Spinal instability, determined by the SINS, was found in 61.7% of the 65–79 years age group and 79.4% of the older group. A significant deterioration in neurological condition and functional status, as indicated by MS and KPI, was observed. The older group demonstrated higher levels of functional impairment as defined by KPI (80 years and older: 47.8% ± 19.5; 65–79 years: 69.0% ± 23.9; *p* < 0.001). A comprehensive display of patient characteristics is provided in Table 1.

**Table 1:** Baseline characteristics

Characteristic	65–79 y *n* = 65	≥ 80 y *n* = 34	*p*-value
Age, years (mean, SD)	68.9 (3.8)	83.4 (3.4)	**< 0.001**
Sex (*n*, %)			0.066
Male	49 (75.4)	21 (61.8)	
Female	16 (24.6)	13 (38.2)	
Body mass index, kg/m^2^ (mean, SD)	26.3 (4.3)	25.0 (2.6)	0.146
Comorbidities			
Age-adjusted CCI score (mean, SD)	5.1 (1.6)	9.2 (2.1)	**< 0.001**
Arterial hypertension (*n*, %)	21 (32.3)	30 (88.2)	**< 0.001**
Myocardial infarction (*n*, %)	7 (10.8)	19 (55.9)	**< 0.001**
Coronary heart disease (*n*, %)	5 (7.7)	14 (41.2)	**< 0.001**
Atrial fibrillation (*n*, %)	5 (7.7)	11 (32.4)	**< 0.001**
Heart failure (*n*, %)	2 (3.1)	12 (35.3)	**< 0.001**
COPD (*n*, %)	8 (12.3)	9 (26.5)	0.171
Diabetes mellitus type II (*n*, %)	6 (9.2)	12 (35.3)	**0.004**
Renal failure (*n*, %)	10 (15.4)	13 (38.2)	**0.020**
Liver disease (*n*, %)	1 (1.5)	6 (17.6)	**0.001**
Gastrointestinal ulcer (*n*, %)	3 (4.6)	6 (17.6)	**0.017**
TIA/stroke (*n*, %)	2 (3.1)	10 (29.4)	**< 0.001**
Malignancy (*n*, %)	38 (58.4)	21 (61.8)	**0.002**
Dementia (*n*, %)	0 (0.0)	3 (8.8)	**0.010**
Previous spinal surgery (*n*, %)	3 (4.6)	3 (8.8)	0.316
ASA class (*n*, %)			0.055
II	21 (32.3)	1 (2.9)	
III	40 (61.5)	5 (14.7)	
IV	4 (6.2)	21 (61.8)	
V		6 (17.6)	
Origin of metastasis (*n*, %)			0.077
Prostate	19 (29.2)	11 (32.4)	
Breast	10 (15.4)	6 (17.6)	
Lung	18 (27.7)	6 (17.6)	
Multiple myeloma	3 (4.6)	5 (14.7)	
Others	15 (23.1)	6 (17.6)	
Location of metastasis (*n*, %)			0.487
Thoracic	44 (61.7)	28 (82.4)	
Lumbar	21 (32.3)	6 (17.6)	
Radiological signs of myelopathy (*n*, %)	31 (47.7)	15 (44.1)	0.692
Spinal Instability neoplastic score (*n*, %)			0.115
0–6	20 (30.8)	7 (20.6)	
7–12	45 (69.2)	27 (79.4)	
ESCC scale (*n*, %)			**< 0.001**
1a	0 (0.0)	0 (0.0)	
1b	0 (0.0)	0 (0.0)	
1c	1 (1.5)	3 (8.8)	
2	48 (73.8)	8 (23.5)	
3	16 (24.6)	23 (67.6)	
Preoperative MS (mean, SD)	81.6 (14.2)	78.2 (16.4)	0.242
Preoperative KPI (mean, SD)	69.0 (23.9)	47.8 (19.5)	**< 0.001**
Frankel Grade			0.105
A	9 (13.8)	11 (32.4)	
B	13 (20.0)	7 (20.6)	
C	31 (47.7)	9 (26.5)	
D	12 (18.5)	7 (20.6)	
E	0 (0.0)	0 (0.0)	

Bold *p*-values indicate statically significant findings

*ASA*, American Society of Anesthesiologists; *CCI*, Charlson comorbidity index; *COPD*, chronic obstructive pulmonary disease; *ESCC*, epidural spinal cord compression; *KPI*, Karnofsky Performance Index; *MS*, motor score of the American Spinal Injury Association grading system; *SD*, standard deviation; *TIA*, transient ischemic attack

### Surgical procedures

Table 2 presents the details of surgical procedures. The duration of decompression was significantly shorter in patients aged 80 and above compared to those aged 65–79 years (148.8 ± 62.5 min vs. 199.4 ± 78.9 min; *p* = 0.004). Moreover, more spinal levels required decompression in the 65–79 years group (2.5 ± 1.0 levels vs. 1.7 ± 0.8 levels; *p* = 0.004). The octogenarian group experienced significantly greater blood loss (535.0 ± 400.1 vs. 143.6 ± 206.1; *p* < 0.001). No significant differences were noted between the groups in terms of LOS or ICU stay. In-hospital and 90-day mortality rates were similar between the groups (in-hospital mortality: 65–79 years 7.7% vs. 80 years and older: 8.8%; *p* = 0.707; 90-day mortality: 65–79 years 18.4%vs. 80 years and older: 14.7%; *p* = 0.526), with reported deaths unrelated to the surgical procedure but attributed to disease progression. Patients aged 65–79 years demonstrated better recovery in terms of functional status (KPS: 79.5% ± 12.7 vs. 58.5% ± 16.2; *p* < 0.001) and degrees of disability (FG: *p* = 0.034). The post-surgery average improvement in the FG was 6.4 points for patients aged 65–79 years, compared to 3.2 points for octogenarians (*p* = 0.002). This suggests that both age groups experienced improvements, but the younger cohort showed a more pronounced recovery than their older counterparts.

**Table 2:** Peri- and postoperative surgical characteristics and clinical course of patients aged 65–79 years and ≥ 80 years who underwent decompression surgery

Characteristic	65–79 y *n* = 65	≥ 80 y *n* = 34	*p*-value
Surgical duration, minutes	199.4 (78.9)	148.8 (62.5)	**0.004**
Number of levels decompressed	2.5 (1.0)	1.7 (0.8)	**0.004**
Estimated blood loss, ml	143.6 (206.1)	535.0 (400.1)	**< 0.001**
Hospital stay, days	10.1 (6.6)	9.5 (5.2)	0.138
ICU stay, days	0.7 (1.7)	0.9 (1.8)	0.497
Mortality			
In-hospital (*n*, %)	5 (7.7)	3 (8.8)	0.707
90-day (*n*, %)	12 (18.4)	5 (14.7)	0.526
30-day readmission (*n*, %)	4 (6.2)	1 (2.9)	0.477
MS	87.7 (12.2)	85.4 (15.7)	0.898
Karnofsky Performance Index	79.5 (12.7)	58.5 (16.2)	**< 0.001**
Postoperative Frankel Grade (*n*, %)			**0.034**
A	1 (1.5)	6 (17.5)	
B	11 (16.9)	5 (14.7)	
C	25 (38.5)	11 (32.4)	
D	28 (43.1)	12 (35.3)	

Except where otherwise indicated, values are mean (SD)

Bold *p*-values indicate statically significant findings

*ICU*, intensive care unit; *MS*, motor score of the American Spinal Injury Association grading system

The overall median survival time was 14.1 months ± 4.3 months (3 days to 24.5 months). At 1-year follow-up, there was no significant difference in survival rates between the groups (15.8 SD 5.1 for ages 65–79 years vs. 12.9 SD 1.2 for those over 80, *p* = 0.085). The mean follow-up period was 26.4 ± 9.4 months with no additional surgery required to address secondary instability. Post-surgery, improvements were observed in both MS and KPS (Table 3). Of the 26 patients, with an average age of 72.4 ± 6.1 years, 11 were octogenarians who survived for more than 2 years post-surgery. These individuals underwent full tumor removal, resulting in a postoperative KI of 67 ± 10%. All patients were treated with radiation targeting the operated segment. Chemotherapy was administered based on the tumor type. Specifically, three patients with lung carcinoma were given immunotherapy, while two with breast cancer additionally received hormone therapy. Except of patients who died during hospitalization all patients received radiation of the operated segment.

**Table 3:** Occurrence of adverse events

Event (%)	65–79 y *n* = 65	≥ 80 y *n* = 34	*p*-value
Deep wound infection	5 (7.7)	3 (8.8)	0.778
Acute respiratory failure	4 (6.2)	2 (5.9)	0.487
Acute heart failure	3 (4.6)	1 (2.9)	0.556
Acute renal failure	2 (3.1)	1 (2.9)	0.422
Septic shock	1 (1.5)	1 (2.9)	0.988
Pneumonia	6 (9.2)	3 (8.8)	0.487
Pleural effusion	1 (1.5)	3 (8.8)	0.599
Ileus	1 (1.5)	4 (11.8)	0.689
Urinary tract infection	6 (9.2)	3 (8.8)	0.487

### Complications and risk factors

Superficial wound infection was the most common complication in both groups, with a prevalence of 8.8%. Octogenarians were more likely to suffer from ileus, while pneumonia was more frequent in the younger group. Detailed information regarding postoperative complications can be found in Table 4. The unique risk factors for mortality included worse functional status and presence of comorbidities, whereas motor weakness, surgical duration, extension of surgery, hospital or ICU stay, and complications did not show a significant correlation (Table 5).

**Table 4:** Comparison of baseline (before surgery) and discharge neurological condition and functional status scores

	65–79 y *n* = 65	65–79y *n* = 65	*p*-value	≥ 80 y Baseline *N* = 34	≥ 80 y Discharge *N* = 34	*p*-value
MS	81.6 (14.2)	87.7 (12.2)	**< 0.001**	78.2 (16.4)	85.4 (15.7)	**0.001**
KPI	69.0 (23.9)	79.5 (12.7)	**< 0.001**	47.8 (19.5)	58.5 (16.2)	**0.002**

All data are presented as mean (SD)

Bold *p*-values indicate statically significant findings

*MS*, motor score of the American Spinal Injury Association grading system; *KPI*, Karnofsky Performance Index

**Table 5:** Risk factors associated with mortality

Risk factor	OR (% 95 CI)	*p*-value
Age	1.2 (1.1–2.3)	0.907
Age-adjusted CCI score	1.8 (1.1–5.1)	**0.017**
Preoperative MS	1.1 (1.0–1.9)	0.140
Preoperative KPI	1.2 (1.1–2.5)	**0.030**
Duration of surgery	1.1 (1.0–1.4)	0.284
Estimated blood loss	1.5 (0.9–2.5)	0.924
Number of levels decompressed	2.5 (1.4–5.6)	0.734
Length of ICU stay	0.5 (0.2–1.0)	0.505
Length of hospital stay	1.1 (0.8–1.5)	0.576
Complications	1.5 (1.2–3.5)	0.928

Bold *p*-values indicate statically significant findings

*CCI*, Charlson Comorbidity Index; *CI*, confidence interval; *ICU*, intensive care unit; *KPI*, Karnofsky Performance Index; *MS*, motor score of the American Spinal Injury Association grading system; *OR*, odds ratio

## Discussion

When managing MESCC, the primary role of surgery is to restore and preserve neurological function. Therefore, decompression surgery may be necessary in certain cases. The treatment of patients presenting with potentially unstable spines is particularly challenging due to the lack of established guidelines. The surgical approach largely hinges upon the individual surgeon’s expertise. It is noteworthy that elderly patients constitute a distinctive cohort, as their pre-existing health conditions may impose an elevated risk in the surgical management of MESCC. However, as of yet, there is a paucity of definitive evidence either contradicting or endorsing surgical intervention for this age group in the context of MESCC.

The present study compares the clinical course of MESCC in patients aged 65–79 years and those aged 80 years and above, who underwent decompression surgery on a potentially unstable spine within 24 h due to the occurrence of acute neurological deterioration. Our results demonstrated that octogenarians exhibited a significantly poorer baseline reserve, with cardiac diseases, diabetes mellitus, and renal failure being more prevalent compared to their younger counterparts. Interestingly, patients from both age groups exhibited similar degrees of motor weakness, as determined by the MS. However, octogenarians were found to have significantly higher grades of disability. Post-surgery, both age groups showed marked improvement in motor functions and functional impairment, although younger patients experienced a more rapid improvement in quality of life. Notably, the duration and extent of surgery were significantly shorter in octogenarians. Mortality rates were comparable between the groups, ranging from 7.7 to 15.0%. We found that the degree of preoperative functional impairment and the rate of comorbidities were significant predictors of mortality, while neither age nor the specific surgical procedures were significant predictors.

In the present study, octogenarians exhibited a mean Charlson Comorbidity Index (CCI) of 9.2, indicating a high degree of comorbidities, whereas the younger group showed significantly lower comorbidity rates (mean CCI 5.1). This suggests that the presence of these compounded factors may argue against surgical interventions in advanced age, even in the case of acute neurological deterioration. A retrospective analysis of 1266 patients with MESCC included 51 patients over 80 years old. Despite presenting worse American Society of Anesthesiologists (ASA) scores and baseline functional status, urgent surgery yielded satisfactory clinical outcomes, leading to an improvement in quality of life [13]. Notably, half of these patients underwent surgery in a palliative setting to preserve neurological function. However, even in these circumstances, postoperative outcomes were comparable to those of younger patients. These results imply that surgical intervention may be a crucial factor in preserving or regaining motor function and ambulatory status, even in such a debilitated cohort. In line with these aforementioned studies, Gao et al. conducted a retrospective analysis involving 55 older patients with a mean age of 78.5 years (range 75–88 years). Their findings also supported the notion that surgery led to a significant increase in quality of life. Although over 90.0% of patients died within a mean time of 18.5 months, the cause of death was attributed to disease progression rather than the surgery itself [14]. Consistent with the studies mentioned above, our findings also suggest that surgery in older patients, including octogenarians, results in substantial improvements in motor function and disability grades. However, younger patients tend to recover more quickly and regain ambulatory status after surgery, with Karnofsky Performance Status (KPS) reaching almost 80.0%.

It is noteworthy that we found both the surgical duration and the extent of the surgery to be significantly longer in patients within the 65–79 age bracket as compared to octogenarians. One plausible explanation for this observation could be that surgical decision-making for older patients primarily balances two key factors: preservation of neurological function and the execution of a surgical procedure that ensures adequate decompression of the epidural space. Importantly, this must be accomplished with a procedure that minimizes intraoperative and perioperative risks, often resulting in the selection of a simple surgical decompression. Interestingly, the intraoperative blood loss was considerably lower in the younger cohort, at 143.6 ml, compared to the octogenarians, who had an average blood loss of 550 ml. This divergence may be attributable to the distinct baseline health statuses of these age groups. Octogenarians were more likely to have a history of significant cardiac diseases, such as myocardial infarction or atrial fibrillation, with 58.8% of them being on anticoagulant therapy prior to surgery. Although reversal agents were administered preoperatively, we noticed an increased propensity for diffuse bleeding during surgery. While reversal agents were administered preoperatively, the efficacy of such agents can sometimes be incomplete or delayed, especially among older individuals [15]. Furthermore, the inherent physiological changes that accompany aging could play a crucial role in this observed disparity. One notable change is the reduced vascular elasticity and increased vascular fragility as described by Greenwald [16]. This inherent fragility might render the vessels more susceptible to intraoperative injury. Moreover, the nutritional and health status of patients, especially among the elderly, can also significantly influence surgical outcomes. It’s been highlighted that elderly patients might exhibit diminished levels of clotting proteins, potentially due to malnutrition or other comorbidities. Such a phenomenon has been detailed by Sobotka et al. [17], indicating a potential impediment to efficient hemostasis during surgery. Additionally, age-associated changes in the inflammatory response might indirectly affect coagulation pathways. Franceschi and Campisi have pointed out that aging is intricately linked with alterations in inflammation, which can, in certain situations, contribute to an augmented risk of surgical bleeding [18]. Concurrently, the implications of other medications should not be overlooked. Many elderly patients, particularly those with cardiac ailments, are often on antiplatelet regimens. Michelson has detailed how these medications, even when not directly anticoagulative, can accentuate perioperative bleeding [19]. Collectively, these factors offer a comprehensive overview of the potential reasons behind the increased blood loss observed in our octogenarian patients. We recognize the importance of delving deeper into these aspects and will work diligently to incorporate and reference them more thoroughly in our manuscript’s discussion section.

We noticed considerable neurological improvements post-surgery in both patient cohorts, potentially due to immediate surgical response to new motor deficits, which significantly affect ambulation [7]. Swift intervention within 48 h of symptom onset significantly improves chances of mobility recovery. Accelerated deterioration of motor function may suggest rapid tumor growth, leading to irreversible spinal cord damage [20]. For older patients, existing degenerative changes could slow postoperative recovery [6, 21, 22]. The observed absolute increments in KPS and MS across both age groups indicate a tangible benefit from the surgical intervention irrespective of age. However, the relative disparities between the groups in the postoperative period, with younger patients achieving notably higher average KPS scores, suggest that the baseline health and resilience of the younger cohort facilitated a more robust overall functional recovery, even if the magnitude of improvement was comparable between groups. This difference is reflective of the intrinsic physiological advantages and greater physiological reserves often associated with a younger age [23]. It is essential to consider the baseline differences in health statuses between the two groups, which could have influenced post-surgical recovery dynamics. The younger cohort had a more favorable preoperative status, as showed by the CCI, enabling them to reach a higher functional ceiling postoperatively. Regarding the varied follow-up periods, we concur that differences in follow-up durations can potentially influence the outcomes. For instance, a longer follow-up might reveal plateauing or even regression in functional scores due to age-related decline or other unrelated health events.

It is crucial to highlight that there was a distinct disparity in complication rates across the two age demographics. Pneumonia emerged as the most widespread complication in patients within the age bracket of 65–79 years, while ileus was more common in individuals aged above 80 years. Supporting these observations, studies conducted by Amelot et al. and Itshayek et al. discovered analogous patterns, with wound complications and pneumonia being the most recurrent. Interestingly, none of these studies necessitated revision surgery, consistent with the findings of our current study [13, 24]. Moving to the aspect of survival rates, Liang et al.’s retrospective analysis of patients over 60 years diagnosed with MESCC disclosed median survival rates of 15 months. However, it is essential to note that this study did not detail the surgical procedures undergone by these patients [25]. Their cohort underwent both simple decompression surgery and combined approaches, yet the potential influence of these surgeries on survival rates was not comprehensively explored. Echoing these results, Amelot et al. documented overall survival rates of approximately 13.9 months, irrespective of the age factor. Importantly, their research underscored that surgical interventions did not correlate with an increased mortality rate among octogenarians in the first year of follow-up when juxtaposed against their younger counterparts [13]. Complementary survival rates were established by Gao et al.’s research, which cited an average survival time of approximately 18 months among elderly patients [14]. In parallel with these aforementioned studies, we observed an overall median survival duration of 14.1 months in our research. Significantly, there were no discernible disparities in survival rates between the groups at the 1-year follow-up (15.8 SD 5.1 for ages 65–79 years vs. 12.9 SD 1.2 for those over 80, *p* > 0.005). This suggests that age may not necessarily pose a contraindication for surgery, even at a more advanced stage. Despite these findings, it is pertinent to note that our patient cohort underwent only a simple decompression procedure with tumor debulking, which resulted in reduced surgical durations. Consequently, the survival rates observed might have been higher than anticipated. Nonetheless, our regression analysis reaffirms that the primary determinants for surgery predominantly hinge on the preoperative functional status of the patients and the prevalence of comorbidities, rather than directly correlating with the patient's age.

### Limitations

A noteworthy merit of our present research is its pioneering nature; it is the first to delve into the outcomes associated with elderly patients aged between 65 and 79 and those 80 or above who transitioned to a surgical intervention. However, it is crucial to recognize certain limitations inherent to our study. Primarily, our study involved a relatively modest patient cohort, which might confine the scope of our conclusions. Nevertheless, given the paucity of extensive research concerning this age group, we believe our findings provide a valuable real-world perspective on the disease landscape. Secondly, our study lacked a younger control group. Thirdly, the potential for selection bias cannot be entirely ruled out given the retrospective design of our study. This may have influenced the selection of patients, their treatments, and how their outcomes were assessed. Detailed information on adjuvant therapies is in most of the cases not available since the vast majority of patients underwent therapies to their close oncological centers and is beyond of the scope of the present study. However, considering the primary goal of the following study, we feel that the information provided is adequate to infer conclusions on outcomes. One of the limitations of this study is the specific focus on a subgroup of patients who presented with pure epidural compression and were treated exclusively with laminectomy. We acknowledge the absence of discussion regarding separation surgery, which is recognized for its ability to effectively decompress ventral compression, particularly in the thoracic spine [26]. While laminectomy offers certain advantages and is less invasive, separation surgery, despite its higher morbidity, may provide superior outcomes in specific contexts. The omission of this surgical approach might may influence clinical decision-making for practitioners seeking holistic insights on various surgical options. In future studies, we will aim to juxtapose these surgical techniques to provide a more nuanced understanding of their respective benefits and drawbacks in managing spinal epidural compression.

Thus, it becomes essential to pursue larger, randomized investigations to corroborate these findings conclusively and delineate the precise attributes of suitable candidates who may reap potential benefits from surgical intervention, even at an advanced age.

## Conclusions

The findings of this research underscore the importance of ongoing education for both general practitioners and spinal surgeons to enhance patient management pathways, mitigate procedural delays, and ensure swift multidisciplinary consultation for patients diagnosed with MSCC. This investigation highlights that age should not serve as a contraindication to spine surgery, particularly when there is a definitive clinical rationale for the operation. Surgical interventions can significantly augment the functional status of patients, maintaining acceptable complication rates. While it is undeniable that older patients with MESCC may have shortened survival rates, an improved quality of life can be attainable through timely diagnosis and immediate treatment intervention. Thus, the swift application of these insights in clinical practice could greatly enhance patient outcomes, even in advanced age groups.

## Data Availability

The datasets generated during and/or analyzed during the current study are available from the corresponding author on reasonable request.
